# Implementing Volume-targeted Ventilation to Decrease Hypocarbia in Extremely Low Birth Weight Infants during the First Week of Life: A Quality Improvement Project

**DOI:** 10.1097/pq9.0000000000000398

**Published:** 2021-05-05

**Authors:** Uduak S. Akpan, Sunny Patel, Paige Driver, Dmitry Tumin

**Affiliations:** From the * Department of Pediatrics, Brody School of Medicine, East Carolina University, Greenville, N.C.; † Department of Pediatrics, Vidant Medical Center, Greenville, N.C.

## Abstract

Hypocarbia in neonates increases the risk of poor neurodevelopmental outcomes. Volume-targeted ventilation (VTV) is associated with decreased hypocarbia and other respiratory morbidities. We noticed a high incidence of hypocarbia in extremely low birth weight (ELBW; <1,000 g) neonates in our Neonatal Intensive Care Unit. Thus, we undertook a quality improvement project to decrease the incidence of hypocarbia (the occurrence of PCO_2_ < 35 mm Hg) in ELBW neonates during the first week of life by 50% over 1 year.

**Methods:**

Our major interventions were employing VTV as the primary mode of mechanical ventilation in neonates less than 28 weeks of gestation or ELBW at birth and increasing staff knowledge regarding hypocarbia. The baseline period spanned May–August 2016. We implemented the interventions in October 2016 and tracked the use of VTV and the incidence of hypocarbia during the first week of life for 12 months.

**Results:**

We analyzed data on 28 and 77 patients in the baseline and postintervention periods, respectively. The use of VTV increased from 39% to 65%. However, the incidence of hypocarbia was not reduced (57% preintervention vs. 64% postintervention). In the postintervention cohort, the incidence of hypocarbia was comparable between VTV and other modes (60% vs. 70%; 95% confidence interval: −32%, 12%; *P* = 0.367), but we noted decreased blood gas sampling and earlier extubation in the VTV group (*P* = 0.002 and *P* = 0.046, respectively).

**Conclusions:**

Successfully increasing VTV in our Neonatal Intensive Care Unit did not decrease hypocarbia during the first week of life. However, we observed the safety of VTV and obtained other desirable results.

## INTRODUCTION

Mechanical ventilation, a frequently necessary life-sustaining therapy in the Neonatal Intensive Care Unit (NICU), could result in ventilator-associated lung injury.^1^ Volume, rather than pressure, is the principal determinant of this condition. Lung damage occurs even after only a few breaths with excessive tidal volume (TV)^2^; hence, early lung-protective strategies such as volume-targeted ventilation (VTV) are encouraged.^3^ Traditionally, time-cycled, pressure-limited ventilation was used in neonates, mainly because older ventilators could not deliver the tiny TVs required to ventilate neonatal lungs.^4^ This obstacle is surmountable using modern ventilators, meaning VTV is now feasible for ventilating neonates.^5,6^ Advantages of VTV are myriad, including decreased hypocarbia and other respiratory morbidities^7–9^ Hypocarbia in extremely low birth weight (ELBW) neonates places them at higher risk for cerebral vasoconstriction and decreased cerebral oxygen delivery, which leads to the development of periventricular leukomalacia, and poor neurodevelopmental outcomes.^10,11^ Decreased likelihood of hypocarbia during VTV results from the autoregulation of peak inspiratory pressures required to deliver a set TV, ensuring a minimum of excessively large breaths (or insufficient volume breaths).^12^

In our NICU, 86% of infants born at 22–29 weeks of gestation require mechanical ventilation. The incidence of hypocarbia in a sample population of mechanically ventilated ELBW neonates was 57%, compared to 6%–46% reported in other units.^11,13^ We inferred from these numbers that our ELBW neonates are at high risk for poor neurodevelopmental outcomes from hypocarbia. Bearing in mind the mechanism of action of VTV, we carried out a quality improvement (QI) project to decrease hypocarbia in ELBW infants. Our specific aim was to decrease the incidence of hypocarbia (a single occurrence of PCO_2_ < 35 mm Hg) in any ELBW neonate by 50% during the first week of life over one year. The advantages of VTV in neonates have been previously described, but to our knowledge, this is the first report of the implementation of VTV in a NICU as part of a QI process.

## METHODS

### Setting

Our NICU is a 50-bed unit that admits approximately 100 ELBW neonates yearly. After initial resuscitation in the delivery room, we initiate a nasal continuous positive airway pressure trial using the Neopuff T-piece resuscitator (Fisher & Paykel Healthcare, Irvine, Calif.). We intubate neonates who are apneic or show poor respiratory effort. In the NICU, we mechanically ventilate intubated neonates, preferring pressure controlled ventilation (PCV)—either conventional or high frequency (oscillator or jet) according to provider discretion. Our blood gas targets reflect permissive hypercapnia with PCO_2_ of 45–60 mm Hg (5.9–7.9 kPa) and pH of 7.25–7.35. The frequency of blood gas sampling mostly depends on the patient’s acuity, but the type of ventilator could also influence it. On average, blood gas sampling in our unit occurs 4–6 times daily. The sampling method is via an umbilical artery catheter, but if insertion is unsuccessful, we use capillary blood sampling via heel sticks. We do not use noninvasive methods of CO_2_ monitoring. In 2015, we introduced VTV using the Draeger Babylog VN500 system (Draeger Inc., Telford, Pa.) into our unit. Contrary to the PCV mode, frequent blood gas sampling in VTV mode is unnecessary because the ventilator autoadjusts the settings as lung compliance changes.^1,5,14^ Sampling can be decreased as long as the neonate is stable and clinically comfortable.

### Intervention

**Fig. 1.**
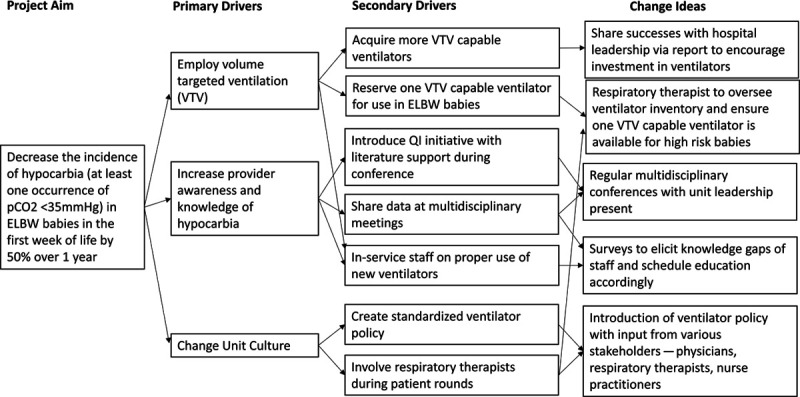
Key driver diagram.

We formed a team comprising a medical student, a neonatology fellow, a neonatology nurse practitioner, a respiratory therapist, and an attending neonatologist. We targeted neonates less than 28 weeks of gestation or weighing less than 1,000 g at birth for this project. These neonates are the most likely to require mechanical ventilation and experience the most severe complications of prematurity. As VTV is associated with stabilization of PCO_2_ levels, we selected increasing the use of VTV as the primary mode of mechanical ventilation for eligible babies immediately after birth as one of our key drivers of change (Fig. 1).

Neonatal VTV works best in patient-triggered modes supporting all inflations,^15^ ensuring the same support level for each patient breath. The set respiratory rates in these modes are effectively backup rates, which are essential if a patient cannot trigger enough breaths spontaneously. A backup rate that is too high prevents adequate triggering by the neonate.^16^ In our NICU, we employ VTV in the assist control mode. We outlined initial ventilator settings as a targeted TV of 4–6 ml/kg, peak end-expiratory pressure of 5–6 mm Hg, and a backup respiratory rate of 30–40 breaths/min.^15^ We decided on these settings after a thorough review of the existing VTV literature. Criteria for changing to PCV included failure to maintain acceptable ventilation and oxygenation on the settings mentioned earlier, persistent respiratory distress, or excessive PIP (>25 cm H_2_O to achieve acceptable PCO_2_ levels). Another primary driver we selected was the education on the proper use of VTV for all NICU practitioners responsible for ventilator management.

### Data Collection

We collected preintervention (baseline) data retrospectively from May to August 2016. In September 2016, we provided an educational session to the NICU staff in the form of an in-service, to ensure proper use of the ventilators. Although there was no official policy in place, we established guidelines for managing ELBW babies in the first week of life with consensus among NICU providers to institute VTV as the first-line management, starting in October 2016.

After analyzing the first month of data, we identified that an insufficient number of VTV-capable ventilators in our unit was a significant challenge to meeting our goals. Thus, we decided to strategically reserve one of these machines to ensure availability if an ELBW neonate was born and required mechanical ventilation. The rationale was to give priority to ELBW neonates due to their high risk for severe morbidities. The hospital purchased additional ventilators that significantly alleviated this problem in April 2017.

To assess staff knowledge and comfort with VTV following the initial education provided in October 2016, we distributed a survey to the NICU providers in January 2017. Based on the survey responses, we provided a repeat educational session in February 2017. This time, in addition to a formal in-service, we devoted several days to small group demonstrations at the bedside, ensuring wider dissemination of the information. Throughout this period, we ensured ongoing education during daily rounds and via conferences to increase staff comfort with interpreting ventilator data and correlating that with the patient’s clinical picture. We completed 2 Plan-Do-Study-Act cycles in addition to the initial cycle, with the following small changes achieved—strategic reserving of VTV capable ventilators due to inadequate numbers and additional education following the staff survey. We eventually introduced a ventilator management policy in the last month of the project.

### Measures

#### Outcome Measure

The percentage of ventilated neonates who experienced hypocarbia in the first week of life. We defined hypocarbia as a blood gas with a PCO_2_ <35 mm Hg (4.67 kPa).^9,11^

#### Process Measures

The process measures were as follows: (1) the percentage of ELBW babies with VTV as the initial mode of ventilation and (2) the percentage of NICU staff who report having received adequate education on the use of VTV after the formal training session. We hypothesized that starting at least 50% of the eligible babies on VTV would reduce our hypocarbia rates by half. Therefore, our goal for the first measure was 50%. We provided a unit-wide training session for staff responsible for managing ventilators to increase their comfort with VTV and counter beliefs regarding the lack of efficacy of VTV in small or very immature neonates. We conducted a survey afterward. Of the 3 groups surveyed, 60% of the physicians and 50% of the respiratory therapists reported that they had received adequate education, whereas only 25% of the nurse practitioners reported being adequately educated. We completed another round of staff education to enhance education on this topic but did not repeat the survey to demonstrate further improvement.

#### Balancing Measure: Rate of Failed Extubation within the First Week of Life

The literature review suggests that neonates ventilated via VTV are likely to be extubated earlier than babies on PCV. We tracked this measure to ensure that higher rates of failed extubation were not associated with early intubation. Failed extubation could occur several times in the hospital course of one neonate. However, we chose to assess only the first episode.

We also chose to track variables that we termed value measures, including the number of blood gases drawn and extubation rates within the first week of life. These value measures are essential because, apart from decreasing hypocarbia, added benefits of VTV will be instrumental in changing the unit culture.

### Data Analysis

We collected project data monthly until September 2017, with data recorded daily for each eligible patient over the first 7 days of life. We included patients in the analysis if they were started on mechanical ventilation at any point within the first week of life. We excluded a total of 19 patients in the post-intervention period—6 who died within the first week of life, and 13 who were initially started on VTV and switched to a PCV mode within the first week of life. We excluded babies who died within the first week due to incomplete data. We used P-charts to track the monthly proportion of babies whose first mechanical ventilation mode was VTV and to monitor the monthly proportion of babies with hypocarbia during the first week of life.^17^ We aimed to have at least 50% of eligible patients started on VTV, with no more than 30% experiencing hypocarbia (representing a decrease of about 50% from baseline). In further analysis, we compared patient characteristics, the incidence and timing of hypocarbia, the duration of the first mechanical ventilation course, the number of blood gas samples drawn, and the need for reintubation according to the initial ventilation strategy. We used rank-sum tests, Chi-square tests, or Fisher’s exact tests, as applicable. We performed data analysis in Stata/SE 15.1 (StataCorp, LP, College Station, Tex.).

### Ethics

The Institutional Review Board at our institution deemed this project exempt from review as it was considered a QI project.

## RESULTS

**Fig. 2.**
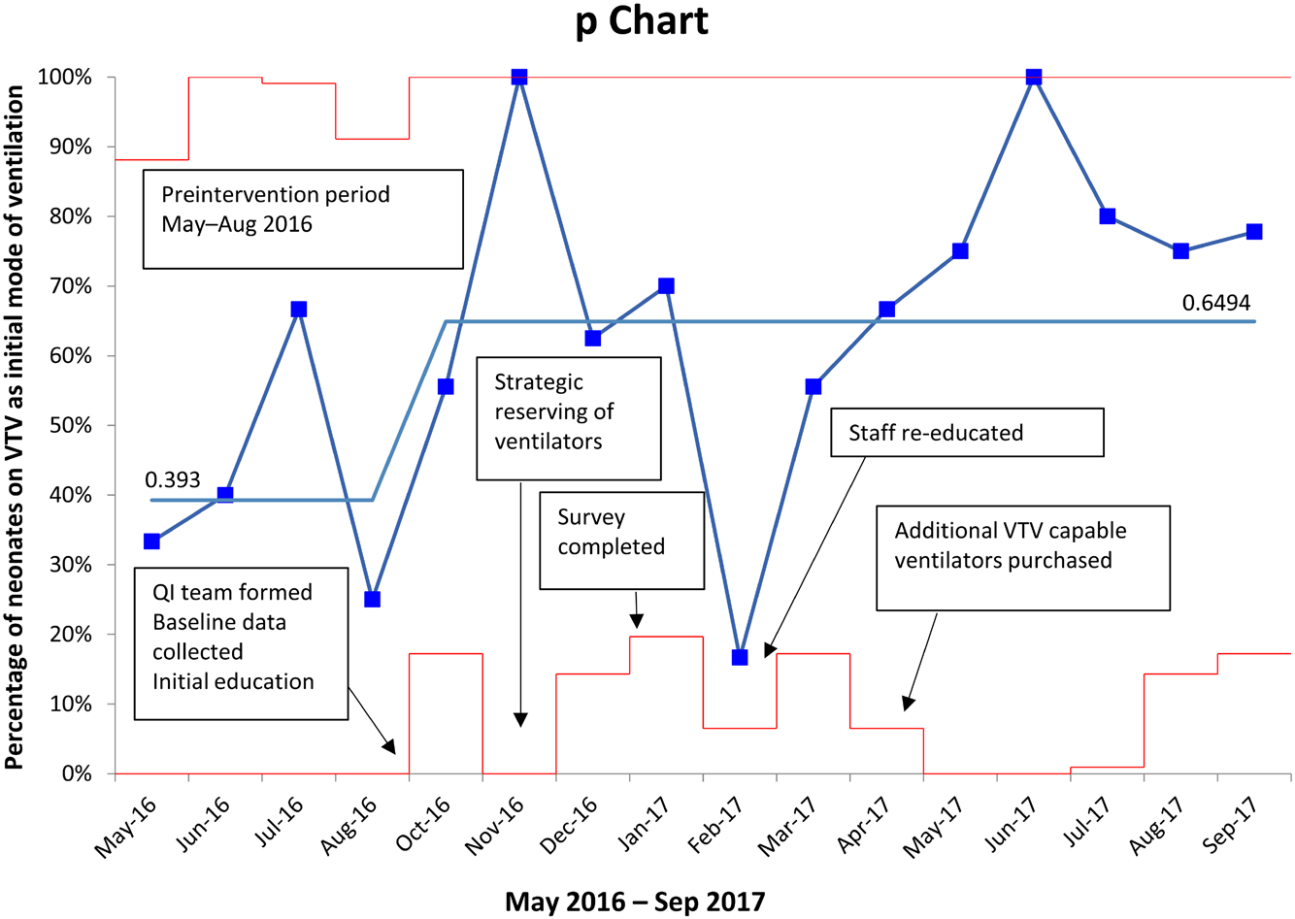
Control chart of neonates with volume-targeted ventilation as the initial mode of ventilation.

We analyzed data from 28 patients during the baseline period (May–August 2016) and 77 patients during the intervention period (October 2016–September 2017). We summarized the characteristics of the baseline and intervention cohorts in Table 1. The groups were similar except for chorioamnionitis prevalence, which was higher in the baseline group (29% vs. 11%, Chi-square *P* = 0.024). During the baseline period, unit providers chose ventilator modes based on device availability, the provider’s comfort, and generally accepted unit practice, resulting in VTV as the initial ventilation mode in 39% of cases. After the project’s start, VTV use increased to 65% of cases over 12 months (Fig. 2). The centerline shift in Figure 2 demonstrates that we exceeded our goal of at least 50% use of VTV in eligible neonates. The remaining patients were started on high-frequency oscillation, high-frequency jet, or synchronized intermittent mandatory ventilation.

**Table 1.** Characteristics of Patients in Baseline and Postintervention Cohorts

Characteristic	Baseline (N = 28) Median (IQR) or N (%)	Intervention (N = 77) Median (IQR) or N (%)
Gestational age (wks)	24 (24, 26)	26 (25, 27)
Birth weight (g)	735 (605, 910)	828 (700, 930)
Delivery by cesarean section	16 (57%)	46 (60%)
Betamethasone × 2 doses	10 (36%)	35 (45%)
Surfactant doses	2 (1, 2)	2 (1, 2)
Chorioamnionitis	8 (29%)	8 (11%)
Apgar score		
1 min	2 (1, 5)	3 (2, 5)
5 min	6 (3, 7)	6 (4, 7)
Age at intubation (d)	0 (0, 0)	0 (0, 0)

In January 2017, a survey sent out to the NICU providers revealed that respondents understood the project rationale and agreed that VTV was an appropriate initial mode of ventilation for ELBW infants. Most of the physicians reported knowing how to use VTV properly. However, a fair number of respiratory therapists and nurse practitioners surveyed desired further education. Therefore, we provided a repeat education session in February 2017. The hospital purchased additional VTV capable ventilators in April 2017, and subsequently, the use of VTV increased to over 70% for the remainder of the project.

**Fig. 3.**
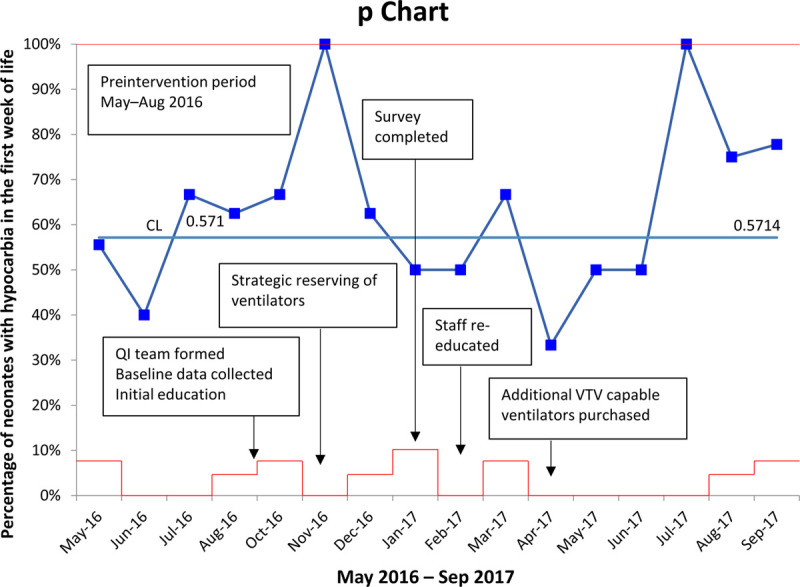
Control chart of hypocarbia incidence in the first week of life while on mechanical ventilation.

Although we achieved and maintained significant improvement in the use of VTV as the first mode of ventilation throughout the project, we did not find a concomitant reduction in the incidence of hypocarbia. During the baseline period, 57% of patients had at least 1 hypocarbia episode in the first week of life, shown as the centerline in Figure 3. Hypocarbia incidence was lowest in June 2016, although this data point may have been skewed by this month having the fewest cases in the baseline period. In the postintervention period, 64% of patients had at least 1 hypocarbia episode in the first week of life. However, the data did not meet the criteria for a centerline shift. After project interventions, performance improved through June 2017 but then began to degrade in the last three months of data (especially in July 2016). A review of data from this period revealed no discernible objective cause for worse performance, such as patient acuity or change in unit practice.

During the postintervention period, we found that patients in the VTV group were slightly less likely to experience hypocarbia (Table 2), but this difference did not reach statistical significance (60% vs. 70%; 95% confidence interval [CI] of difference −32%, 12%; *P* = 0.367). There was no statistically significant difference between the groups regarding the timing of the earliest hypocarbia episode or the number of hypocarbia episodes in the first week of life. Patients in the VTV group had a significantly shorter time to extubation (median of 1 vs. 7 d; *P* = 0.046) but were more likely to require reintubation within the first week of life (36% vs. 11%; *P* = 0.030). These patients also experienced decreased blood gas sampling (median of 8 vs. 18 total blood gas samples in the first week; *P* = 0.002).

**Table 2.** Patient Characteristics and Outcomes by Initial Mode of Mechanical Ventilation in Postintervention Cohort

Characteristic or Outcome	VTV (N = 50) Median (IQR) or N (%)	Other mode (N = 27) Median (IQR) or N (%)	*P*
Gestational age (wks)	26 (25, 27)	25 (24, 27)	0.054
Birth weight (g)	850 (745, 950)	770 (670, 910)	0.115
Delivery by cesarean section	32 (64%)	14 (52%)	0.300
Betamethasone × 2 doses	23 (46%)	12 (44%)	0.896
Surfactant doses	1 (1, 2)	2 (1, 2)	0.519
Chorioamnionitis	6 (12%)	2 (7%)	0.704
Apgar score			
1 min	3 (2, 5)	3 (1, 5)	0.495
5 min	7 (4, 7)	6 (3, 7)	0.350
Age at intubation (d)	0 (0, 0)	0 (0, 0)	0.505
Duration of first intubation (d)*	1 (0, 3)	7 (0, 7)	0.046
Total number of gas samples in first 7 d of life	8 (3, 15)	18 (4, 28)	0.002
Reintubation within 7 d of life	18 (36%)	3 (11%)	0.030
Patients with hypocarbia within 7 d of life	30 (60%)	19 (70%)	0.367
Timing of first hypocarbia episode†	1 (1, 1)	1 (1, 1)	0.153
No. hypocarbia episodes	1 (0, 2)	1 (0, 3)	0.349

*Observations censored at 7 d of life.

†Day of life, among cases with at least 1 hypocarbia episode.

IQR, interquartile range.

## DISCUSSION

There was a significant increase in the use of VTV in our NICU, as identified by the centerline shift in Figure 2. Still, we did not find a reduction in hypocarbia commensurate with the increase in VTV use over the postintervention period. Secondary analysis revealed a decreased likelihood of hypocarbia using VTV, although this did not reach statistical significance. Time to first extubation was shorter in the patients started on VTV after birth. However, there was also a significant risk of failed extubation. Patients on VTV were larger and more mature than those on other modes of ventilation, principally the high-frequency oscillator. This observation aligns with many providers’ belief that VTV is not effective in small or very immature neonates.^18^ Our results demonstrate that ELBW infants can tolerate ventilation modes other than high-frequency modes; only 21% of those started on VTV failed that mode and transitioned to high-frequency ventilation.

The TV delivered by a ventilator depends in part on the patient’s lung mechanics. VTV is advantageous over PCV modes in that the ventilator autoadjusts the pressure required to deliver a set TV, resulting in consistent TVs and decreased hypocarbia risk. Although our QI project meaningfully increased VTV use in our NICU over one year, the incidence of hypocarbia remained roughly the same. A few reasons may explain this observation. First, tracking the duration rather than merely noting the incidence of hypocarbia may be necessary to demonstrate the differences between ventilation modes. Second, providers may not have been sufficiently familiar with VTV and could not employ optimal settings on the ventilator. The TV required for normocapnia is inversely proportional to birth weight, being about 6 ml/kg in babies smaller than 500 g and around 5 ml/kg in babies larger than 700 g.^19^ If the set TV is inadequate, tachypnea occurs as the infant tries to accommodate this inadequacy.^20^ The blood gas evaluation will show hypocarbia that may be erroneously interpreted as extubation readiness, leading to premature extubation. This phenomenon could explain the likelihood of earlier extubation, but higher failed extubation rates noted in the VTV group. The rate observed (36%) is in keeping with the published figure for extremely preterm infants.^21,22^ We countered this problem with staff education on correlating data from the ventilator, blood gas, and clinical examination whenever a failed extubation occurred. The cumulative duration of mechanical ventilation contributes more to respiratory morbidity than repeated intubations, with increased risk of bronchopulmonary dysplasia reportedly occurring only in infants exposed to 4 or more courses of mechanical ventilation.^23^ Thus, despite the risks associated with reintubation, earlier extubation is encouraged.^24,25^ Last, as the project progressed, there was an increased awareness of hypocarbia in general, causing providers to be more attentive to weaning pressures on the PCV modes of ventilation. This trend may explain the similarity in the incidence of hypocarbia between the patient groups in our project, a finding that differs from other studies in the literature.^8,9^

We encountered several obstacles in carrying out this project. Change in the unit culture was the greatest. We found that the value measures, notably decreased blood sampling, were crucial factors that ensured staff buy-in. We addressed the limited number of VTV-capable ventilators by reserving a volume ventilator for target neonates until additional ventilators were acquired.

A limitation of our project may have been the use of the incidence of hypocarbia as the primary patient outcome. Hyperventilation causing hypocarbia could occur as a compensatory measure for metabolic acidosis in extremely preterm babies. Mild hypocarbia, not leading to alkalosis, may be tolerable. Evaluation of pH, PCO_2,_ and the fluctuation of these factors may be required to assess the impact of volume ventilation and the risk for neurodevelopmental problems reliably.^26^ A further limitation was the short duration of the baseline data collection period and outlier observations during this period, which potentially limited the statistical precision of outcomes measured in this phase of the project. Moreover, our inferential statistics may have been limited by the nonrandom assignment of patients included in the analysis. However, results for crucial project outcomes were consistent with inferential statistics or statistical process control rules to describe the outcome of the project interventions. In the postintervention period, similar patient outcomes between ventilator modes raise the question of whether ventilator mode superiority can be reliably demonstrated in premature infants, which is unfortunately outside the scope of the present project.

Our QI project was useful because it increased awareness of hypocarbia in our NICU, the avoidance of which is essential for improved neurodevelopmental outcomes. Even though there was no decrease in the incidence of hypocarbia noted during the duration of this project, the secondary benefits experienced during this project, including earlier extubation and decreased blood gas sampling, are important clinical outcomes in the care of premature neonates.

## DISCLOSURE

The authors have no financial interest to declare in relation to the content of this article.

## ACKNOWLEDGMENTS

We would like to thank Dr. Sergio Golombek for his input toward the final manuscript, Lauren Jones NNP, and Darian Brewington, RRT, for their help in carrying out the project.
